# A Case of Syncope Due to Intracardiac Leiomyomatosis

**DOI:** 10.7759/cureus.22666

**Published:** 2022-02-27

**Authors:** Shrinjaya Thapa, Bipin Ghimire, Paras Thapa, Tucker Billups

**Affiliations:** 1 Internal Medicine, Beaumont Hospital, Royal Oak, USA; 2 Internal Medicine, Kathmandu Medical College, Kathmandu, NPL

**Keywords:** intracardiac leiomyomatosis, syncope, leiomyoma, atrial mass, intravenous leiomyomatosis

## Abstract

We present a case of a 46-year-old female presenting with syncope. Echocardiography initially showed a right atrial mass. Further evaluation revealed a mass arising from the fundus of the uterus, with a tumor thrombus in the left gonadal vein, extending into the left renal vein and through the inferior vena cava (IVC) into the right heart across the tricuspid valve. She was managed with surgical resection, and postoperative pathology was consistent with intravenous leiomyomatosis (IVL). IVL is a rare uterine smooth muscle cell neoplasm which extends into the venous system. Gynecological tumors are often overlooked in differential diagnosis for atrial masses. A benign tumor like fibroid, in rare circumstances, can extend into the right side of the heart causing dynamic obstruction to outflow tract, thus increasing mortality. The objective of this article is to present such a case and highlight the broad differentials of atrial masses, including IVL.

## Introduction

Primary cardiac tumors are rare, with an incidence of less than 0.1% [1]. They include benign tumors like myxoma, lipoma, and papillary fibroelastoma as well as malignant tumors like sarcoma, rhabdomyosarcoma, lymphoma, and pericardial mesothelioma. Secondary tumors are 20 times more common than primary tumors [1]. They metastasize through hematogenous spread, lymphatic spread, direct invasion or intracavitary diffusion through the vena cava into the heart [2]. Tumor thrombus extending to the right heart is commonly seen with renal cell carcinoma. Less common etiologies include intravenous leiomyoma, gynecological malignancies, Wilm’s tumor, hepatoma, and adrenocortical carcinoma [2].

This article was previously presented as a poster at the ACP Michigan Regional meeting on October 14, 2021.

## Case presentation

A 46-year-old female with a history of hypertension, hyperlipidemia, pre-diabetes, morbid obesity, and uterine fibroid presented to the hospital for two episodes of syncope on the day prior to arrival. During the first episode, she was sitting up in a chair when her eyes rolled back in her head and was unresponsive for about 15 secs as witnessed by her spouse. During the second episode, she was making breakfast when she suddenly lost consciousness and fell forward on the countertop; regaining consciousness shortly after. She denied biting her tongue, post-ictal confusion, bowel or bladder incontinence, pre-syncopal symptoms, palpitations, headache, neck pain, vomiting, diarrhea, or similar episodes previously. Surgical history was significant for dilation and curettage for menometrorrhagia. She was not on any oral contraceptive or hormonal therapy.

In the emergency department, blood pressure was 146/94, pulse 91/min, respiration 21/min, oxygen saturation 96% and orthostatic vitals were negative. There were no pertinent physical examination findings, including the absence of a murmur on cardiac auscultation. Complete blood count, comprehensive metabolic panel, D-dimer, and troponin were within normal limits. Chest X-ray and CT scan of her head did not show any abnormal findings. An electrocardiogram showed sinus tachycardia with low voltage QRS complex. She was resuscitated with intravenous fluids, placed on telemetry, and admitted to the observation unit. 

**Figure 1:**
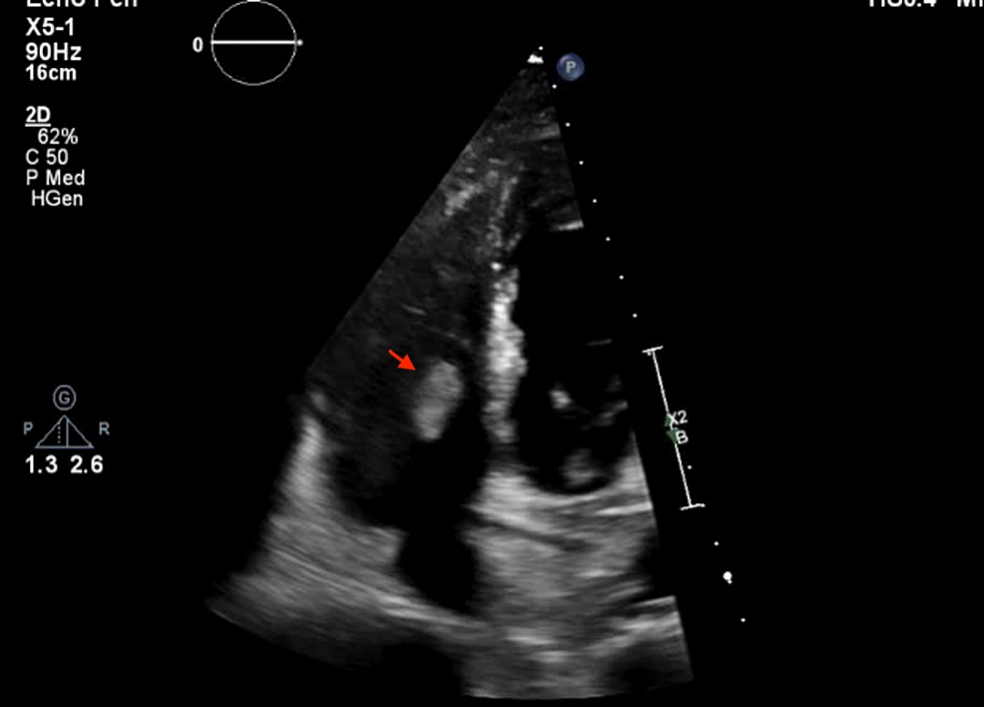
2D transthoracic echocardiography Mobile echogenic structure in the right atrium with trace tricuspid regurgitation.

**Figure 2:**
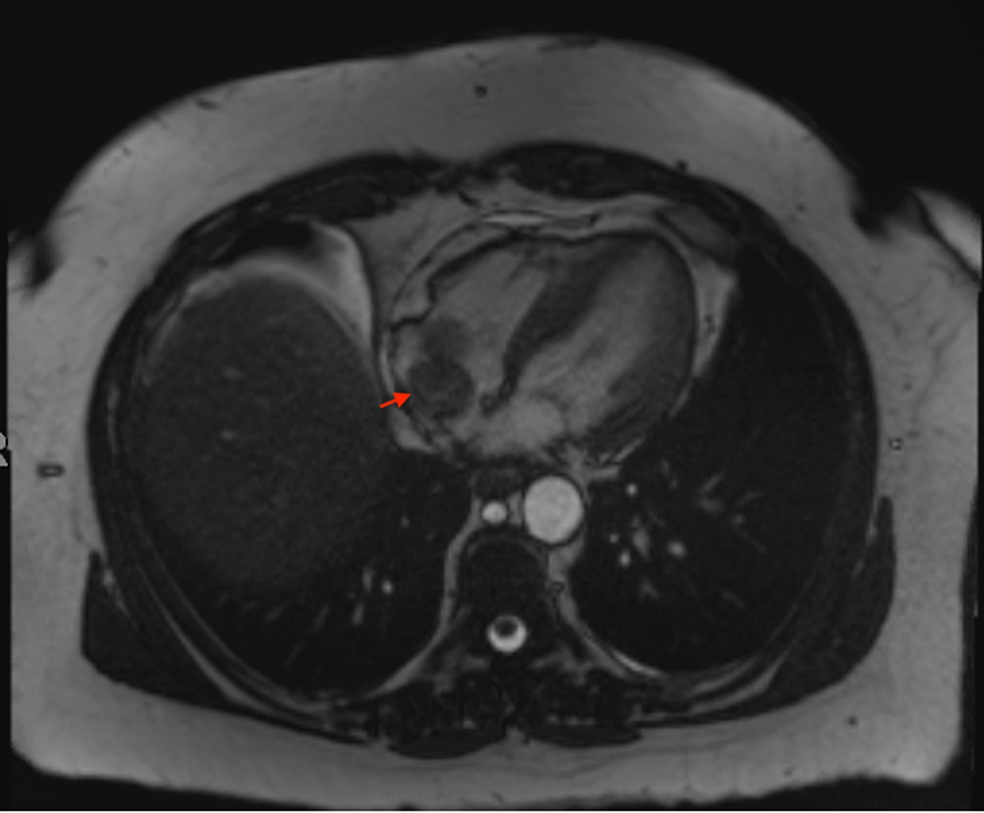
MRI of the heart with gadolinium A mobile mass is seen within the right heart, in the right atrium, and the right ventricle. The intra-cardiac portion of the mass measures 5 x 1.8 cm.

**Figure 3:**
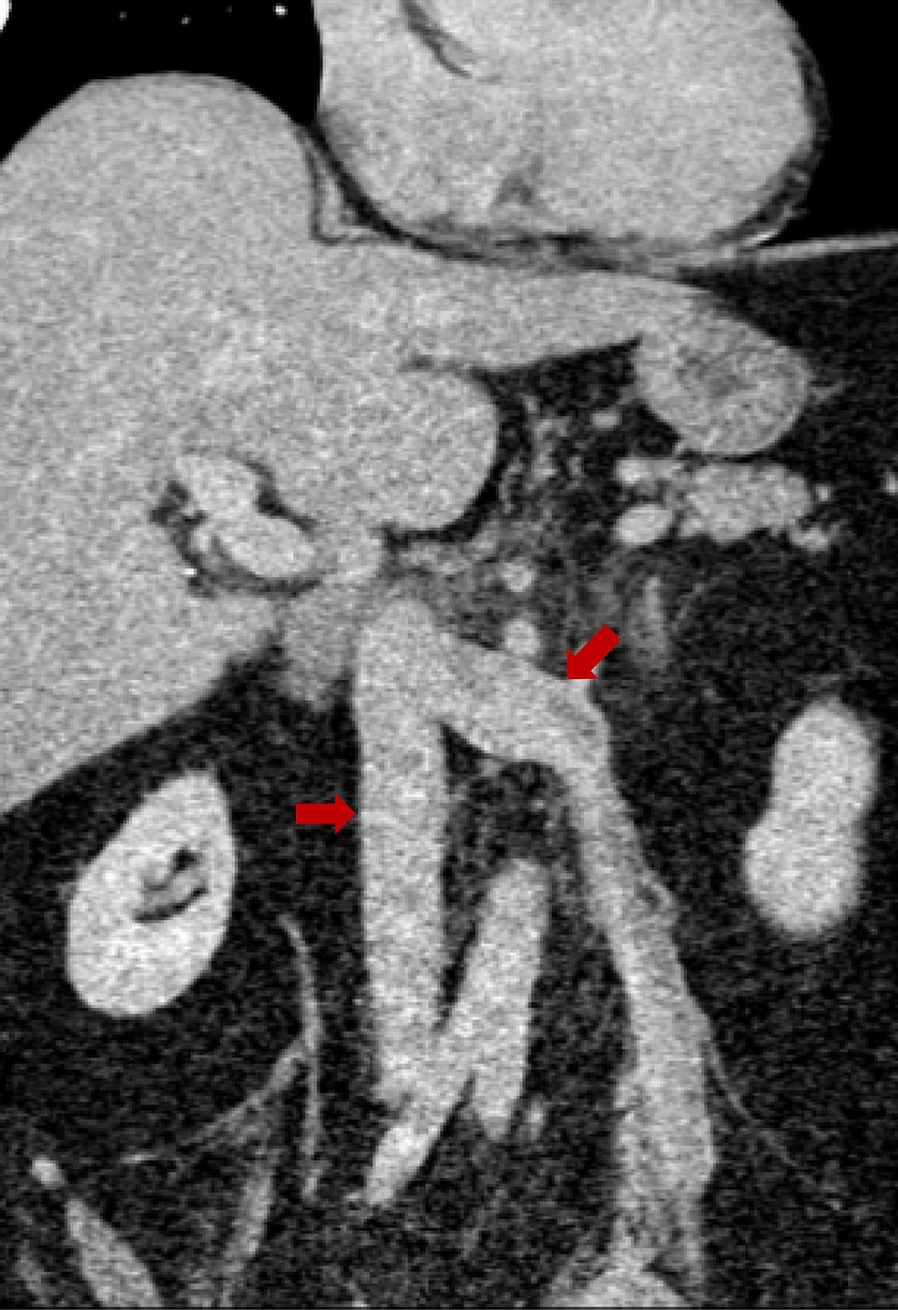
Coronal contrast-enhanced CT of the abdomen Continuous hypodense lesion extending from the left gonadal vein, through the left renal and into the IVC. Hypodensity in the right atrium, shown more clearly in the cardiac MR appears to be a continuation of the lesion.

An echocardiogram revealed a mobile echogenic mass in the right atrium extending from the inferior vena cava (IVC) and moving across the tricuspid valve, causing dynamic obstruction of the right atrial and tricuspid flow (Figure 1). She was promptly started on intravenous heparin for possible thrombus. CT angiography of the chest was negative for pulmonary embolism. Cardiac MRI and CT abdomen/pelvis with contrast showed a mass originating from the renal vein, extending through the IVC into the right heart across the tricuspid valve terminating into the right ventricle (Figures 2-3) The intra-cardiac part of the mass measured 5 cm x 1.8 cm. 

Abdominal/pelvic MRI showed a heterogenous enhancing mass arising from the fundus of the uterus measuring 13.4 x 10.9 x 7.6 cm, inseparable from the left ovary but favored to be of uterine origin. There was tumor thrombus in the left gonadal vein, extending into the left renal vein and through the IVC, into the heart. Pre-surgical differential diagnosis included atypical leiomyoma, sarcoma, or metastatic ovarian tumor. She was transferred to a higher center for surgery, and underwent a single-stage operation which included exploratory laparotomy with total abdominal hysterectomy/bilateral salpingo-oophorectomy by gynecology, left gonadal vein resection, and tumor thrombectomy with vascular reconstruction per urology-oncology, and removal of the right atrial and ventricular tumor along with IVC tumor with cardiopulmonary bypass by cardiothoracic surgery. Pathology of the vena cava excised mass revealed tumor thrombus consistent with intravenous leiomyomatosis (IVL). Uterine pathology consisted of leiomyomata with liomatous differentiation and IVL. Complete resection of the tumor was achieved and no anti-estrogen therapy was offered.

She was admitted for a total of 15 days between two hospitals. The postoperative course was complicated by elevated glucose and high insulin requirement. She was started on amiodarone 200 mg twice daily and enoxaparin 40 mg daily for prophylaxis for a month. She was discharged on oral pain medication and close follow-up as an outpatient. CT chest/abdomen/pelvis in three-month intervals did not show any recurrence of the disease. The plan is to monitor for disease recurrence every six months for two yrs since the time of diagnosis, and consider anti-estrogen therapy if she has a recurrence. She remains disease-free for a year and a half since diagnosis.

## Discussion

IVL is a rare uterine neoplasm comprised of benign smooth muscle cells, which by extension grows within the intrauterine and extrauterine venous system [3]. It was first described in 1896 by Birch-Hirschfeld, reported to be present only in 0.097% of genital smooth muscle tumors [3]. There have been two main theories regarding the pathogenesis of this disease process, with one suggesting it arises from the smooth muscles on the walls of the veins in the myometrium, while the other theory supports the direct vascular invasion of the myometrium into the venous circulation. Norris and Parmley provide evidence for both these theories [4]. Furthermore, Lam et al. reported that IVL extends into the systemic venous circulation via the uterine vein and less commonly through the ovarian vein [5]. Presentation of IVL depends on the extent and size of tumor thrombus, however, a particularly deadly presentation is that of intracardiac leiomyomatosis (ICL). ICL is present in 10%-30% of IVL, with the risk of mortality significantly increased once it crosses the tricuspid valve. Extension to the right ventricle may result in outflow tract obstruction, and involvement of the pulmonary artery may develop pulmonary embolism [6].

IVL exclusively occurs in women, mostly peri-menopausal with a median age of 43 years [3]. There is a wide range of presentation of IVL depending on the location involved, ranging from asymptomatic, abdominal pain, irregular bleeding, abdominal mass, lower extremity swelling, ascites, and hepatosplenomegaly. Li et al. conducted a comprehensive review of 194 cases of ICL and reported the most common presenting symptoms as dyspnea, syncope, lower extremity edema, and palpitations [6].

Pre-operative diagnosis is mainly through imaging. Echocardiography revealing a right-sided cardiac mass without attachment to the endocardium or endothelial surface, originating and freely moving in the IVC without a stalk, in a woman with a history of hysterectomy or leiomyoma should raise suspicion for ICL [7]. MRI of the abdomen/pelvis should be obtained next, which gives precise information about the tumor location and its characteristics. This is followed by CT with angiography, which gives detailed information regarding the complete path of the lesion. This can be seen as worm-like tumor extension through the ovarian or uterine vein, into the IVC, and ultimately into the right heart [8]. Complete surgical resection, when possible, is the treatment of choice to prevent a recurrence. A multi-disciplinary approach in either a single-stage or two-stage operation is required. Involved parties in the surgery include a cardiothoracic surgeon, vascular surgeon, and gynecologist. Incomplete resection will lead to recurrence in about 33.3% of cases, with conflicting evidence regarding the use of anti-estrogen therapies in patients with residual tumor [6].

## Conclusions

Cardiac tumors are rare tumors with secondary tumors being more common than primary. IVL, also a rare gynecological benign smooth muscle neoplasia, can extend all the way to the heart from the uterus and present as a cardiac mass. Hence, ICL should be considered in the differential diagnosis of cardiac tumor, given their significant mortality once it crosses the tricuspid valve. 

## References

[1] Lam KY, Dickens P, Chan AC (1993). Tumors of the heart. A 20-year experience with a review of 12,485 consecutive autopsies. Arch Pathol Lab Med.

[2] Yusuf SW, Bathina JD, Qureshi S (2012). Cardiac tumors in a tertiary care cancer hospital: clinical features, echocardiographic findings, treatment and outcomes. Heart Int.

[3] Du J, Zhao X, Guo D, Li H, Sun B (2011). Intravenous leiomyomatosis of the uterus: a clinicopathologic study of 18 cases, with emphasis on early diagnosis and appropriate treatment strategies. Hum Pathol.

[4] Norris HJ, Parmley T (1975). Mesenchymal tumors of the uterus. V. Intravenous leiomyomatosis: a clinical and pathologic study of 14 cases. Cancer.

[5] Lam PM, Lo KW, Yu MY, Wong WS, Lau JY, Arifi AA, Cheung TH (2004). Intravenous leiomyomatosis: two cases with different routes of tumor extension. J Vasc Surg.

[6] Li B, Chen X, Chu YD, Li RY, Li WD, Ni YM (2013). Intracardiac leiomyomatosis: a comprehensive analysis of 194 cases. Interact Cardiovasc Thorac Surg.

[7] Li R, Shen Y, Sun Y (2014). Intravenous leiomyomatosis with intracardiac extension: echocardiographic study and literature review. Tex Heart Inst J.

[8] Nakai G, Maeda K, Yamamoto K (2015). Uterine Intravenous Leiomyomatosis with Cardiac Extension: Radiologic Assessment with Surgical and Pathologic Correlation. Case Rep Obstet Gynecol.

